# Preliminary Study on the Effect of *Bacillus amyloliquefaciens* TL on Cecal Bacterial Community Structure of Broiler Chickens

**DOI:** 10.1155/2019/5431354

**Published:** 2019-10-03

**Authors:** Yuxuan Hong, Yingxian Cheng, Yanjuan Li, Xiaowen Li, Zutao Zhou, Deshi Shi, Zili Li, Yuncai Xiao

**Affiliations:** ^1^College of Veterinary Medicine, Huazhong Agricultural University, Wuhan, China; ^2^State Key Laboratory of Agricultural Microbiology, Huazhong Agricultural University, Wuhan, China; ^3^Key Laboratory of Preventive Veterinary Medicine in Hubei Province, Huazhong Agricultural University, Wuhan, China

## Abstract

Probiotics can promote the health and growth performance of animals through modulation of intestinal microbiota. When used as a feed additive, they have the potential to minimize or abolish the use of antibiotics. In this study, we investigated the effect of the probiotic strain *Bacillus amyloliquefaciens* TL on the growth performance and cecum microflora composition in Cobb 500 broiler chickens. In total, 180 broilers were randomly divided into three groups—each group comprised 4 pens, and each pen contained 15 chickens. The three groups were fed either a control diet, or a diet supplemented with either the antibiotic chlortetracycline or *B. amyloliquefaciens* TL. Broilers were weighed, and cecum contents were collected on days 7, 14, 21, and 35, respectively. The broilers in both the antibiotic and probiotic groups exhibited significant weight gain compared with controls, exhibiting increases of 16.02% and 13.40%, respectively, after 35 days (*P* < 0.01). Similarly, the feed conversion ratio (FCR, 1–35 days) of broilers in the chlortetracycline and *B. amyloliquefaciens* TL groups was lower than that of the controls. HiSeq high-throughput sequencing of 16S rRNA of the cecal microbiota was performed on days 7, 14, 21, and 35, respectively. The *Firmicutes*/*Bacteroidetes* ratio was higher in the chlortetracycline and *B. amyloliquefaciens* TL groups than in the control group on days 14, 21, and 35, and especially on day 21. The prevalence of genera *Oscillospira*, *Ruminococcus*, *Butyricicoccus*, and *Faecalibacterium* (*Firmicutes*) was higher in the antibiotic and probiotic groups, while that of *Bacteroides*, *Parabacteroides* (*Bacteroidetes*), and *Lactobacillus* was higher in the control group. In this study, the changes in the microbiota of the probiotic group were similar to those in the antibiotic group. These results suggest that the probiotic strain *B. amyloliquefaciens* TL can modulate the cecal microbiota of broilers similar to chlortetracycline.

## 1. Introduction

Since the 1950s, it has become a common practice to add antibiotics to livestock and poultry feed to improve growth and prevent infection by pathogenic microorganisms [1]. Although the EU countries have banned the usage of antibiotics and growth promoters since 2006, some countries still use antibiotics in large quantities in the poultry industry. This extensive use of antibiotics is associated with an increased prevalence of drug-resistant bacterial strains in food, water, and soil [2, 3]. Not only does increased pathogen resistance through the selection of drug-resistant mutants make it more difficult to treat and control the spread of some diseases, but residual drugs within food also pose a potential threat to human health [4]. Chlortetracycline is a broad-spectrum tetracycline antibiotic that is inexpensive and exhibits highly active resistance both to Gram-negative and Gram-positive bacteria; thus, it is commonly used in animal feeds to maintain health and improve growth. Chlortetracycline has been widely used in eight provinces of China [5]. Hence, there is an urgent need to develop new feed additives that can replace antibiotics.

Animal metabolism is a complex process involving pathways that are regulated by the genomes of both the host and the bacteria that comprise the intestinal flora. The intestinal flora thus plays an important role in animal health, growth, and metabolism and in nutrient absorption. The intestinal flora may be regulated through the use of probiotics [6, 7], which may inhibit the growth of pathogenic species [8–12]. Furthermore, probiotics promote a healthy microbial community in the intestines, limit drug residues within food, and help to reduce the spread of drug-resistant microorganisms and may improve the feed conversion rates, making them a suitable alternative to antibiotics.

The Gram-positive strain *Bacillus amyloliquefaciens* is a facultative anaerobic bacterium with the ability to secrete proteases, lipases, and amylases, such as pectinase, glucanase, and cellulase, to enhance digestion efficiency [12, 13]. Previously, lactic acid bacteria were shown to significantly enhance amylase activity in the small intestines of chicks, which may improve overall growth [14]. In addition to efficiently degrading plant carbohydrates, *Bacillus amyloliquefaciens* also resists high temperatures, high pressures, and acidic and alkaline conditions [12, 15]. Studies have shown that increasing the intestinal population of *B. amyloliquefaciens* increased the average daily weight gain of broiler chickens in a linear fashion; furthermore, significant improvements in serum IgA and IgG levels and reduced fecal NH3 and H2S emissions were observed [16, 17]. The result showed that the growth parameters increased directly upon the addition of *B. amyloliquefaciens* to the basal diet. Nevertheless, some studies reported no significant improvement in above parameters of broilers when fed *B. amyloliquefaciens* [18, 19], which may be due to strain differences. The purpose of this study was to evaluate whether the addition of *Bacillus amyloliquefaciens* TL to broiler diets promoted broiler weight gain, explore its effects on chicken gut microbiota using 16S rRNA gene sequencing, and determine whether the strain can be used as a new feed additive to reduce or replace the use of chlortetracycline. The study provides new insights into the role of probiotics in the diet and may help optimize the use of probiotics as feed additives to replace antibiotics, thereby achieving high yields in broilers.

## 2. Materials and Methods

### 2.1. Experimental Additives

The *B. amyloliquefaciens* TL strain and chlortetracycline were obtained from Hubei Huada Real Technology Co. (Wuhan City, Hubei Province, China). We used *B. amyloliquefaciens* TL at a concentration of 200 grams/ton, with an effective viable number of 2.0 × 10^10^ CFU/g; the dose was advised by the product description. Chlortetracycline was added to a final concentration of 50 grams/ton, according to a previous report [20]. The composition and nutrient levels of the basic diets are described in [Supplementary-material supplementary-material-1].

### 2.2. Laboratory Animals

The study was performed in strict accordance with the Guide for the Care and Use of Laboratory Animals Monitoring Committee of Hubei Province, China, and the protocol was approved by the Committee on the Ethics of Animal Experiments at the College of Veterinary Medicine, Huazhong Agricultural University.

In total, 180 one-day-old male Cobb 500 broiler chickens were randomly divided into three groups—each group comprised 4 pens, and each pen contained 15 chickens. The chickens were fed a basal diet, a diet supplemented with the probiotic strain *B. amyloliquefaciens* TL, or a diet supplemented with the antibiotic chlortetracycline, until they were 35 days old. The chicken coop and surrounding areas were disinfected with potassium permanganate and formalin before the trial. The temperature of the chicken coop was maintained at approximately 33°C before the chickens were 7 days old and then gradually reduced to 23°C when the chickens were between 7 and 21 days old. The temperature was maintained at 23°C thereafter. To maintain the health of the chickens, the coop was cleared of manure daily, and the chickens had access to artificial feed available throughout the day and unlimited water via nipple drinkers. In addition, the coop was ventilated using an automated system (Da Mu Ren ventilation equipment, China) to control the opening of the baffled inlet vents and the fan duty cycle depending on indoor temperature. The amount of feed consumption and the residual feed by each group was recorded daily. Body weight was measured on days 7, 14, 21, and 35.

### 2.3. Sample Collection, DNA Extraction, and Pyrosequencing

Eight chickens were sampled from the control group, probiotic group, and antibiotic group on days 7, 14, 21, and 35, respectively. Immediately after euthanasia, the abdominal cavity was exposed, the cecum from each bird was cut open, and the contents were collected in a sterile 5 mL tube, stored on ice, and later frozen and stored at −80°C. In total, 80 samples for cecal contents were collected (the number of samples collected on days 7, 14, 21, and 35 was 4, 7, 8, and 8 for the control group; 4, 7, 8, and 8 for the probiotic group; 4, 7, 7, and 8 for the antibiotic group, respectively).

Total bacterial DNA was extracted from chicken feces by using a QIAamp DNA stool mini kit (Qiagen, Germany) according to the manufacturer's instructions, and NanoDrop 2000 spectrophotometer was used to measure the purity and concentration of the DNA (Thermo, USA) [21]. In brief, PCR was performed to amplify the V4 hypervariable regions of bacterial 16S rRNA genes, using the 515F-806Rr primer set (515F: 5′-GTGCCAGCMGCCGCGGTAA-3′ and 806R: 5′-XXXXXXGGACTA CHVGGGTWTCTAAT-3′) [22]. All PCR was carried out in 30 *μ*L reaction volumes containing 15 *μ*L of Phusion High-Fidelity PCR Master Mix (New England Biolabs, Ipswich, MA, USA), 3 *μ*L forward and reverse primers, 10 *μ*L template DNA, and 2 *μ*L ultrapure water on a Bio-Rad T100 gradient PCR instrument. The following cycling conditions were used: 1 cycle of 98°C for 1 min, 30 cycles of 98°C for 10 s, 50°C for 30 s, and 72°C for 30 s, followed by a final elongation step of 72°C for 5 min. PCR products were detected via electrophoresis using 2% agarose gels. Samples with a bright band of 400–450 bp were chosen for further experiments. Only PCR products without primer dimers and contaminant bands were used for sequencing by synthesis.

Sequencing libraries were generated using a TruSeq® DNA PCR-Free Sample Preparation kit (Illumina, USA) following the manufacturer's recommendations, and index codes were added. The library quality was assessed on the Qubit@ 2.0 Fluorometer (Thermo Scientific) and Agilent Bioanalyzer 2100 system. Finally, the library was sequenced on an Illumina HiSeq platform, and 250-bp paired-end reads were generated by Novogene (Beijing, China) as described [23]. The 16S rRNA gene sequences were submitted to NCBI's Sequence Read Archive (SRA) with the accession number SRP109238.

### 2.4. Statistical Analysis

Paired-end reads were merged using FLASH software package version 1.2.7 (http://ccb.jhu.edu/software/FLASH) [24], and the splicing sequences were called raw tags. Raw sequence data processing was performed using the quality control protocols within the Quantitative Insights Into Microbial Ecology (QIIME) software package version 1.7.0 (http://qiime.org/index.html) [25], and (UCHIME) (http://www.drive5.com/usearch/manual/uchime-algo.html) software was used to remove chimeric sequences and obtain effective tags [26]. The UPARSE software version 7.0.1001 (http://drive5.com/uparse/) was then used to cluster all of the effective tags into operational taxonomic units (OTUs) with 97% identity, determine representative sequences for the OTUs, and annotate them. Further analysis was performed using the Ribosomal Database Project (RDP) Classifier tool and Greengenes database [27–29]. Finally, all of the data for each sample were pooled and normalized for further analysis using the Greengenes database.

R software (Version, 2.15.3) was used to generate the dilution curve and species accumulation curve and to analyze the alpha diversity index between groups, and the Wilcoxon test was conducted. Qiime software (Version 1.7.0) was used to calculate the weighted UniFrac distance and build the unweighted pair-group method with arithmetic means (UPGMA) sample clustering tree. R software (Version 2.15.3) was used to draw the nonmetric multidimensional scaling (NMDS) diagram [30, 31]. Relative abundances of the main phyla and genera (abundance > 1%) were calculated, and MetaStats analysis using the R software at the phylum and genus levels was carried out [32]. The intergroup nonparametric *t*-test was used to analyze differences in community structures between treatment groups. ANOVA with Tukey's test was used to analyze interactions between treatments and growth performance indexes [33]. The results were presented as the mean ± SD, considering *P* value < 0.05 as significant.

## 3. Results

### 3.1. Growth Performance

The average body weight, daily weight gain, and total feed conversion ratio were calculated at the indicated time points for each of the three broiler groups (Table 1). After 7, 21, and 35 days, the average body weight was higher in the probiotic group and antibiotic group than in the control group (*P* < 0.05). The differences on day 21 were particularly striking; the mean body weight and daily weight gain of the probiotic group increased by 25.33% (days 14–21) and 51.47%, respectively, compared with that of the control group (*P* < 0.01). The broilers in both the antibiotic and probiotic groups exhibited significant weight gain compared with controls, exhibiting increases of 16.02% and 13.40%, respectively, after 35 days (*P* < 0.01). Additionally, the FCR (days 1–35) was lower in both the probiotic and antibiotic groups than in the control group.

### 3.2. DNA Sequencing Data Analysis and Quality Control

Fecal samples were collected, and DNA was sequenced. In total, 4,808,967 raw paired-end reads were obtained, and after merging them, 4,743,856 raw spliced tags remained. An average of 56,822 tags per sample was collected. Following quality control, 4,545,779 expressed sequence tags remained, with an average length of 253.1 bp. The average number of taxon tags on days 7, 14, 21, and 35 was 55105, 54161, 55259, and 55019 for the probiotic group; 55158, 56668, 55056, and 53596 for the antibiotic group; and 51350, 57530, 55056, and 56012 for the control group, respectively. The Q20 and Q30 quality scores were 99.4335 and 98.815, respectively, and the effect (%) was 94.522. Good's coverage was at least 99% for each group ([Supplementary-material supplementary-material-1]).

### 3.3. Microbial Abundance and Diversity Analysis

**Figure 1:**
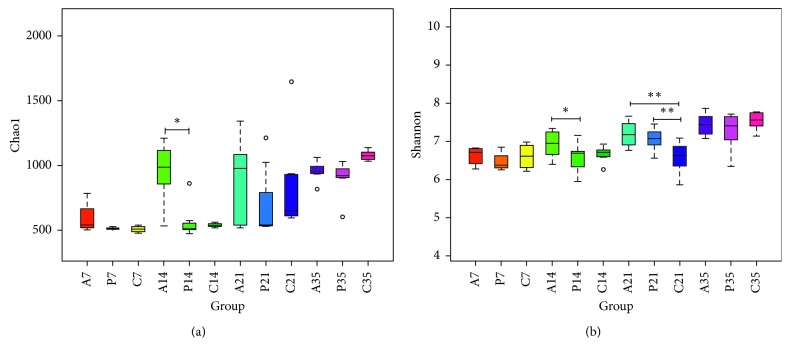
Differences in bacterial community diversity, richness, and cecum microfloral structure following treatment with probiotics (“P”), antibiotics (“A”), or no treatment (“C”). (a) Bar graph showing the Chao1. (b) Shannon index for all treatment groups on days 7, 14, 21, or 35 (^*∗*^*P* < 0.05; ^*∗∗*^*P* < 0.01).

Chao1 indices were selected to identify community richness, and the Shannon index was used to identify community diversity. On day 14, the cecal composition in the antibiotic group was significantly greater than that in probiotic and control groups (Figure 1(a)), and the species diversity in the antibiotic group was significantly greater than that in the probiotic group (*P* < 0.05), as determined by calculating Shannon's diversity index (Figure 1(b)). Furthermore, after 21 days, the species diversity in both the probiotic and antibiotic groups was significantly greater than that in the control group (Figure 1(b); *P* < 0.01).

**Figure 2:**
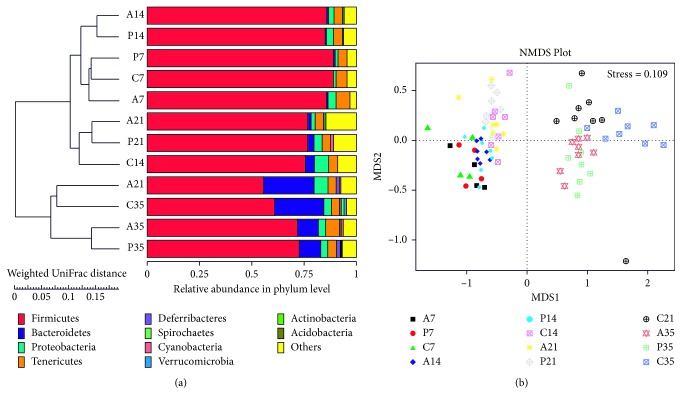
(a) Principal coordinate analysis of the unweighted UniFrac distance metric for all treatment groups on the indicated days. The relative abundance of various phyla is indicated by colored bars. (b) NMDS analysis of the cecal microflora of the indicated groups on the indicated days. Closer samples have more similar species composition (^*∗*^*P* < 0.05; ^*∗∗*^*P* < 0.01).

In this study, NMDS was used to identify differences in microbial community structure among broilers in different treatment groups. The weighted UniFrac distance matrix was chosen for UPGMA cluster analysis to study the similarity between different groups. On day 7, UPGMA cluster analysis showed the antibiotic, probiotic, and control groups to be clustered on the same branch at the phylum level (Figure 1(a)). However, on days 14, 21, and 35, the antibiotic and probiotic groups were clustered on the same branch, with the control group on a different branch. Furthermore, on days 21 and 35, the control groups clustered together on the same branch. Consistent with this finding, NMDS analysis showed that the three groups clustered together after day 7 (Figure 1(b)). After 14, 21, and 35 days, the distance between the antibiotic and probiotic groups was small, although these groups differed from the control group. Similar to the UPGMA cluster analysis, the controls from days 21 and 35 clustered together (Figure 2(b)).

### 3.4. Microbial Community Membership Analysis

**Figure 3:**
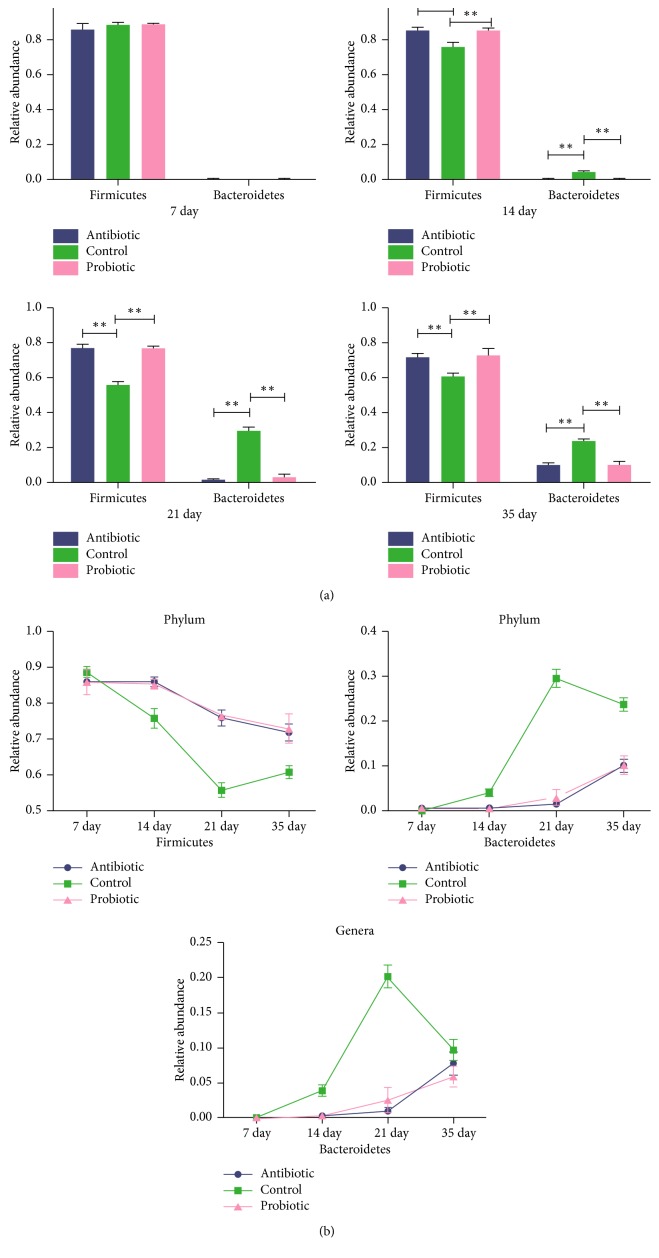
(a) Relative abundance of *Firmicutes* and *Bacteroidetes* in the antibiotic (“A”), probiotic (“P”), and control (“C”) groups at days 7, 14, 21, and 35 is shown. (^*∗*^*P* < 0.05; ^*∗∗*^*P* < 0.01). (b) The prevalence of *Firmicutes* and *Bacteroidetes* (phylum level) and *Bacteroides* (genus level) in the antibiotic and probiotic groups and the control group as time progressed.

A total of 10 phyla were shared by chickens from all groups ([Supplementary-material supplementary-material-1]): *Firmicutes*, *Bacteroidetes*, *Proteobacteria*, *Tenericutes*, *Deferribacteres*, *Cyanobacteria*, *Verrucomicrobia*, *Actinobacteria*, *Acidobacteria*, and *Spirochaetes*, which accounted for 85.5%–96.8% of the total microbiota species present. *Firmicutes* accounted for 76.6% of the species present, and *Bacteroidetes*, *Proteobacteria*, and *Tenericutes* were the next most common gut bacterial phyla, accounting for 7.06%, 3.6%, and 4.4% of the species present, respectively ([Supplementary-material supplementary-material-1]). Although there were no significant differences at day 7, the proportion of *Firmicutes* bacteria in the antibiotic and probiotic groups was significantly higher than that in the control group on days 14, 21, and 35 (*P* < 0.01; Figure 3(a)), and the proportion of *Bacteroidetes* in the control group was significantly higher than that in the other two groups on days 14, 21, and 35 (*P* < 0.01), but there was no significant difference in the relative abundance of *Firmicutes* and *Bacteroidetes* between the antibiotic and probiotic groups. However, as time progressed, the proportions of *Firmicutes* and *Bacteroides* in the three treatment groups changed significantly, especially in the control group. The probiotic and antibiotic groups exhibited similar trends (Figure 3(b)).

At the genus level, a total of 282 genera were identified from all samples. Most genera were shared among the three groups at the same age. On day 7, the main genera in the three groups were not different (Table 2). The proportion of *Bacteroides* and *Bilophila* in the control group was significantly higher than that in the antibiotic and probiotic groups (*P* < 0.05), whereas *Oscillospira* and *Anaeroplasma* were significantly less abundant in the control group than in the other two groups on day 14 (*P* < 0.05; Table 3). On day 21, the genera *Bacteroides*, *Lactobacillus*, *Parabacteroides*, and *Mucispirillum* were significantly more prevalent in the control group than in the antibiotic or probiotic groups (*P* < 0.05), whereas *Oscillospira*, *Butyricicoccus*, *Anaeroplasma*, and *Ruminococcus* were significantly more prevalent in the antibiotic and probiotic treatment groups (*P* < 0.05). Additionally, *Mucispirillum* was significantly more abundant in the probiotic group than in the antibiotic group (*P* < 0.05; Table 4). On day 35, the genera *Parabacteroides*, *Treponema*, and *Bilophila* were significantly more abundant in the control group than in the antibiotic group (*P* < 0.05), and *Faecalibacterium* was significantly more abundant in both the probiotic and antibiotic groups than in the control group (*P* < 0.05). *Mucispirillum* had the highest abundance in the probiotic group (Table 5).

## 4. Discussion

It has been widely known that chlortetracycline administration can improve the health and growth performance of animals. The results of the present study demonstrate that supplementation with chlortetracycline in broiler diets can promote broiler weight gain and daily weight gain and lower FCR. However, our studies also showed that supplementation with probiotic *B. amyloliquefaciens* TL affords similar advantages. Lei and Ahmed showed that the growth performance was improved and FCR was reduced when *B. amyloliquefaciens* was directly added in the basal diet [16, 17]. Another study showed that a diet supplemented with *B. amyloliquefaciens* can increase nutrient utilization, improve growth performance, and balance cecal microflora in chicken [34].

The Gram-positive strain *B. amyloliquefaciens* is a facultative anaerobic bacterium which is closely related to *B. subtilis* [12]. Some studies have shown improvement in broiler growth and reduction of FCR in broilers fed with additives comprising *B. subtilis* [35–40]. However, Jerzsele reported that the addition of probiotics to broiler feed had no effect on broiler growth [19]. These contradictory results may be related to the probiotic strain, dose, and growth period of the test animal.

Some studies have reported that the changes in growth performance of broilers with antibiotics and probiotics are associated with changes in the cecal microbiota [41–43]. This study explored the changes of cecal microbiota after chlortetracycline and *B. amyloliquefaciens* TL supplementation by using 16S rRNA sequencing technology. Good's coverage, quality control, and Q20/Q30 analyses demonstrated that the sequencing results were acceptable. Chao1 and Shannon indexes were used to study the impact of antibiotic and probiotic supplementation on cecal microbiota, mainly on days 14 and 21.

Some studies have suggested that antibiotics modulate the cecum flora in the early stage of growth [41, 42, 44]. UPGMA cluster and NMD analysis in the present study indicated that the bacterial communities of the antibiotic group and probiotic group were similar.

In this study, *Firmicutes* was the most predominant phylum, followed by *Bacteroidetes* and *Proteobacteria*, similar to previous reports [33, 45–47]. On days 14, 21, and 35, the *Firmicutes/Bacteroidetes* ratio was higher in the antibiotic and probiotic groups than in the control group. It has been established that a higher *Firmicutes/Bacteroidetes* ratio promotes broiler growth [33, 42, 46]. A high proportion of *Firmicutes/Bacteroidetes* in the cecum results in a higher fermentation capacity, enabling the fermentation of more volatile fatty acids and thereby promoting fat deposition [48]. A higher *Firmicutes/Bacteroidetes* ratio also plays a role in human weight management, with high values in obese people and low values in lean people [48]. The results shown in Figure 3(b) indicate that, as time progressed, the proportion of *Bacteroidetes* increased, and the proportion of *Firmicutes* decreased, especially in the control group, while this change was slower in the antibiotic and probiotic groups (the trend was similar in these two groups).

At the genus level, the main genera were different as time progressed, but the changes in the probiotic and antibiotic groups remained consistent at similar ages (Tables 2–5). On day 7, the main genera were not significantly different among the three groups, but the proportion of *Escherichia* spp. was reduced in the probiotic group compared with the control and antibiotic groups, similar to the results of a previous study that demonstrated that *B. amyloliquefaciens* TL can inhibit *Escherichia* [16]. On days 14, 21, and 35, the proportions of genera *Oscillospira*, *Ruminococcus*, *Butyricicoccus*, and *Faecalibacterium* (*Firmicutes*) were higher in the antibiotic and probiotic groups. Some reports have indicated that *Firmicutes* can enhance the intestinal absorption of nutrients, resulting in obesity [49, 50]. *Oscillospira* spp. was detected in the rumen of cattle and sheep and in the soft feces of rex rabbits [51, 52], which might indicate that *Oscillospira* is involved in fermentation. *Faecalibacterium* produces butyrate via butyryl-CoA:acetate CoA-transferase with net consumption of acetate, and acetate stimulates its growth on carbohydrate energy sources [53, 54]. *Ruminococcus* can degrade cellulose to increase the absorption of carbohydrate [55–58]. *Butyricicoccus* can stimulate the growth of intestinal epithelial cells [59–61]. *Bacteroides*, *Parabacteroides* (*Bacteroidetes*), and *Lactobacillus* (*Firmicutes*) were abundant in the control groups after day 14. In this study, *Bacteroides* exhibited a striking increase in prevalence from days 14 to 21, and it exhibited high prevalence through day 35 in the control group (Figure 3(b)) compared with the antibiotic and probiotic groups. The daily-weight gain (14–21 days and 21–35 days) and body-weight gain on days 21 and 35 in the control group was significantly lower (*P* < 0.01) than that in the antibiotic and probiotic groups. This indicates that *Bacteroides* proportion is negatively correlated with weight gain in broilers. Some studies have reported that the relative abundance of *Bacteroidetes* increased as obese mice lost weight [49].

## 5. Conclusions

In conclusion, the intestinal microbiota changed with time and diet in the present study. Our results indicate that *B. amyloliquefaciens* TL can modulate the cecal microbiota of broilers, similar to chlortetracycline. The main difference in the probiotic and antibiotic groups compared with the control group was the higher proportion of *Firmicutes* and lower proportion of *Bacteroidetes* at the same age. This composition might be beneficial for the absorption and utilization of nutrients in broilers. These findings indicate that the diet-added probiotic preparation *B. amyloliquefaciens* TL has a similar growth promoting effect as chlortetracycline. The widespread addition of antibiotics to animal feed stimulates growth and rapidly increases productivity. However, while inhibiting harmful bacteria, it also inhibits some beneficial microorganisms and causes antibiotic resistance to become more serious [62]. TL has the potential to be used as a new feed additive to reduce the use of chlortetracycline in the poultry industry, which is important to alleviate the growing problem of antibiotic resistance. It is worth noting that our research is still preliminary. The mechanism of interaction between *B. amyloliquefaciens* TL and the host and its impact on metabolic activities in broilers is the next research plan to further validate the effectiveness of TL in replacing chlortetracycline.

## Figures and Tables

**Table 1:** Growth performance in broiler chickens.

Parameters		Treatment			*P* value		
		C	A	P	P vs. C	A vs. C	P vs. A
		(Mean ± SD)	(Mean ± SD)	(Mean ± SD)			
Body weight (g)	7 days	147.48 ± 11.08	146.50 ± 9.09	157.35 ± 8.58	n.s.	n.s.	^*∗*^
	14 days	499.75 ± 20.67	509.50 ± 46.20	532.50 ± 24.09	n.s.	n.s.	n.s.
	21 days	858.62 ± 30.74	999.75 ± 64.47	1076.12 ± 98.48	^*∗∗*^	^*∗∗*^	^*∗*^
	35 days	1901.38 ± 76.36	2206.00 ± 263.57	2156.12 ± 198.61	n.s.	^*∗∗*^	n.s.

Daily weight gain (g)	0–7 days	15.23 ± 1.38	15.22 ± 1.15	16.82 ± 1.08	^*∗*^	n.s.	^*∗*^
	7–14 days	50.32 ± 1.51	51.86 ± 5.35	53.59 ± 2.33	n.s.	n.s.	n.s.
	14–21 days	51.27 ± 1.74	70.04 ± 3.01	77.66 ± 10.74	^*∗∗*^	^*∗∗*^	^*∗*^
	21–35 days	80.21 ± 3.72	92.79 ± 16.24	83.08 ± 9.23	n.s.	^*∗*^	n.s.

FCR (1–35 days)		2.02	1.63	1.62			

n.s. means not significant (*P* > 0.05); ^*∗*^significant (*P* < 0.05); ^*∗∗*^extreme significant (*P* < 0.01). C = control group; A = chlortetracycline group; P = *Bacillus amyloliquefaciens* TL group; FCR: feed conversion ratio.

**Table 2:** Differences in the abundance of the eight bacterial genera among the control, antibiotic, and probiotic groups, as determined on day 7 (*n* = 4).

Taxa	Relative abundance		
Genus	C	P	A
	(Mean% ± SD)	(Mean% ± SD)	(Mean% ± SD)
*Faecalibacterium*	5.99 ± 0.026	7.57 ± 0.022	8.81 ± 0.034
*Osicillospira*	3.56 ± 0.002	4.11 ± 0.003	4.06 ± 0.003
*Escherichia*	1.03 ± 0.007	0.429 ± 0.002	1.82 ± 0.009
*Butyricicoccus*	2.48 ± 0.013	1.81 ± 0.004	1.52 ± 0.003
*Clostridium*	1.17 ± 0.003	1.26 ± 0.001	1.46 ± 0.004
*Ruminococcus*	1.60 ± 0.007	1.03 ± 0.001	1.23 ± 0.002
*Ruminococcus*	1.58 ± 0.002	1.59 ± 0.002	1.04 ± 0.002
*Anaeroplasma*	1.09 ± 0.004	0.85 ± 0.002	1.10 ± 0.001

None of the comparisons have significant difference on the same line based on the *P* value. C = control group; A = chlortetracycline group; P = *Bacillus amyloliquefaciens* TL group.

**Table 3:** Differences in the abundance of nine bacterial genera among the control, antibiotic, and probiotic groups, as determined on day 14 (*n* = 7).

Taxa	Relative abundance			*P* value		
Genera	C	P	A	P vs. C	A vs. C	P vs. A
	(Mean% ± SD)	(Mean% ± SD)	(Mean% ± SD)			
*Faecalibacterium*	8.3 ± 0.024	15.25 ± 0.039	14.57 ± 0.039	n.s.	n.s.	n.s.
*Oscillospira*	2.94 ± 0.002	3.79 ± 0.003	4.09 ± 0.005	^*∗*^	^*∗*^	n.s.
*Butyricicoccus*	1.88 ± 0.005	2.29 ± 0.002	1.75 ± 0.003	n.s.	n.s.	n.s.
*Anaeroplasma*	0.60 ± 0.001	1.13 ± 0.000	1.30 ± 0.001	^*∗*^	^*∗*^	n.s.
*Ruminococcus*	1.45 ± 0.002	1.37 ± 0.002	1.48 ± 0.002	n.s.	n.s.	n.s.
*Bacteroides*	3.92 ± 0.008	0.32 ± 0.003	0.31 ± 0.001	^*∗*^	^*∗*^	n.s.
*Lactobacillus*	0.68 ± 0.001	0.55 ± 0.002	1.08 ± 0.002	n.s.	n.s.	n.s.
*Clostridium*	1.07 ± 0.002	1.09 ± 0.002	0.96 ± 0.001	n.s.	n.s.	n.s.
*Bilophila*	1.34 ± 0.003	0.020 ± 0.000	0.0015 ± 0.000	^*∗*^	^*∗*^	n.s.

n.s. means not significant (*P* > 0.05); ^*∗*^significant (*P* < 0.05). C = control group; A = chlortetracycline group; P = *Bacillus amyloliquefaciens* TL group.

**Table 4:** Difference in the abundance of nine bacterial genera among the control, antibiotic, and probiotic groups, as determined on day 21 (in the antibiotic group, *n* = 7; in the probiotic and control groups, *n* = 8).

Taxa	Relative abundance			*P* value		
Genera	C	P	A	P vs. C	A vs. C	P vs. A
	(Mean% ± SD)	(Mean% ± SD)	(Mean% ± SD)			
*Faecalibacterium*	7.44 ± 0.010	6.61 ± 0.011	7.56 ± 0.015	n.s.	n.s.	n.s.
*Bacteroides*	20.24 ± 0.016	2.55 ± 0.018	1.02 ± 0.005	^*∗*^	^*∗*^	n.s.
*Oscillospira*	1.34 ± 0.002	3.19 ± 0.004	2.65 ± 0.001	^*∗*^	^*∗*^	n.s.
*Parabacteroides*	2.82 ± 0.008	0.0097 ± 0.010	0.013 ± 0.000	^*∗*^	^*∗*^	n.s.
*Ruminococcus*	0.82 ± 0.000	1.62 ± 0.002	1.69 ± 0.003	^*∗*^	^*∗*^	n.s.
*Butyricicoccus*	0.62 ± 0.002	1.74 ± 0.002	1.51 ± 0.002	^*∗*^	^*∗*^	n.s.
*Mucispirillum*	1.43 ± 0.005	0.117 ± 0.001	0.0125 ± 0.000	^*∗*^	^*∗*^	^*∗*^
*Anaeroplasma*	0.09 ± 0.000	1.21 ± 0.004	1.07 ± 0.004	^*∗*^	^*∗*^	n.s.
*Lactobacillus*	5.11 ± 0.010	0.36 ± 0.002	0.906 ± 0.002	^*∗*^	^*∗*^	n.s.

n.s. means not significant (*P* > 0.05); ^*∗*^significant (*P* < 0.05). C = control group; A = chlortetracycline group; P = *Bacillus amyloliquefaciens* TL group.

**Table 5:** Difference in the abundance of nine bacterial genera among the control, antibiotic, and probiotic groups, as determined on day 35 (*n* = 8).

Taxa	Relative abundance			*P* value		
Genera	C	P	A	P vs. C	A vs. C	P vs. A
	(Mean% ± SD)	(Mean% ± SD)	(Mean% ± SD)			
*Faecalibacterium*	3.29 ± 0.005	8.34 ± 0.015	9.25 ± 0.020	^*∗*^	^*∗*^	n.s.
*Bacteroides*	9.72 ± 0.015	5.93 ± 0.015	7.77 ± 0.016	n.s.	n.s.	n.s.
*Lactobacillus*	5.20 ± 0.005	4.31 ± 0.002	6.91 ± 0.022	n.s.	n.s.	n.s.
*Oscillospira*	2.16 ± 0.002	2.55 ± 0.002	2.17 ± 0.002	n.s.	n.s.	n.s.
*Ruminococcus*	1.24 ± 0.001	1.24 ± 0.001	1.50 ± 0.000	n.s.	n.s.	n.s.
*Parabacteroides*	7.06 ± 0.003	1.49 ± 0.006	0.74 ± 0.003	^*∗*^	^*∗*^	n.s.
*Bilophila*	1.815 ± 0.007	0.375 ± 0.003	0.127 ± 0.001	^*∗*^	^*∗*^	n.s.
*Mucispirillum*	0.84 ± 0.007	1.88 ± 0.011	0.16 ± 0.000	n.s.	^*∗*^	^*∗*^
*Treponema*	1.30 ± 0.001	0.174 ± 0.000	0.32 ± 0.001	^*∗*^	^*∗*^	n.s.

n.s. means not significant (*P* > 0.05); ^*∗*^significant (*P* < 0.05). C = control group; A = chlortetracycline group; P = *Bacillus amyloliquefaciens* TL group.

## Data Availability

The data used to support the findings of this study are available from the corresponding author upon request.

## Supplementary Materials

Supplementary Materials Table S1: composition and nutrient level of basic diets (0–35 days). Table S2: the relative abundance of *Firmicutes*, *Bacteroidetes*, *Proteobacteria*, and *Tenericutes* in broilers treated with chlortetracycline and *Bacillus amyloliquefaciens* TL and in a control group on days 7, 14, 21, and 35. Figure S1: the Good's coverage index of the 80 broilers treated with chlortetracycline and *Bacillus amyloliquefaciens* TL and in a control group on days 7, 14, 21, 35. “A” = chlortetracycline group; “P” = *Bacillus amyloliquefaciens* TL group; “C” = control group. Figure S2: relative abundances at the phylum level of the main bacteria found in the cecum of 80 broiler chicken treated with chlortetracycline and *Bacillus amyloliquefaciens* TL and in a control group on days 7, 14, 21, 35. “A” = chlortetracycline group; “P” = *Bacillus amyloliquefaciens* TL group; “C” = control group.
