# Supplementary material for: Preliminary Study on the Effect of Bacillus amyloliquefaciens TL on Cecal Bacterial Community Structure of Broiler Chickens
Source: Biomed Res Int. 2019 Oct 3;2019:5431354. doi: 10.1155/2019/5431354 (PMC6794981; doi:10.1155/2019/5431354)
Supplement: Supplementary Materials — Table S1: composition and nutrient level of basic diets (0–35 days). Table S2: the relative abundance of Firmicutes, Bacteroidetes, Proteobacteria, and Tenericutes in broilers treated with chlortetracycline and Bacillus amyloliquefaciens TL and in a control group on days 7, 14, 21, and 35. Figure S1: the Good's coverage index of the 80 broilers treated with chlortetracycline and Bacillus amyloliquefaciens TL and in a control group on days 7, 14, 21, 35. “A” = chlortetracycline group; “P” = Bacillus amyloliquefaciens TL group; “C” = control group. Figure S2: relative abundances at the phylum level of the main bacteria found in the cecum of 80 broiler chicken treated with chlortetracycline and Bacillus amyloliquefaciens TL and in a control group on days 7, 14, 21, 35. “A” = chlortetracycline group; “P” = Bacillus amyloliquefaciens TL group; “C” = control group. [file 5431354.f1.pdf]

Table S1. Composition and nutrient level of basic diets (0–35 days)

Ingredient	Mixture ratio	Nutrient name	value
		1-21 days	
Corn	56.00	ME Mcal/kg	3.11
Soybean Meal	30.00	Crude protein%	23.04
Vegetable Oil	4.00	Calcium%	0.99
Premixes	1.00	Lysine%	1.33
Salt	0.30	Methionine%	0.58
Fishmeal	3.20	Methionine+Cystine%	0.94
Limestone	1.20	Available Phosphorus	0.48
Dicalcium Phosphate	1.40		
Corn Gluten Meal	2.50		
Total	100		
		22-35 days	
Corn	63.00	ME Mcal/kg	3.2051
Soybean Meal	23.00	Crude protein%	20.5710
Vegetable Oil	4.20	Calcium%	0.8737
Premixes	1.00	Lysine%	1.1803
Salt	0.20	Methionine%	0.5590
Fishmeal	3.70	Methionine+Cystine%	0.8775
Limestone	1.00	Available Phosphorus	0.4558
Dicalcium Phosphate	1.20		
Corn Gluten Meal	2.30		
Total	100		

Note: Supplied per kilogram of diet: vitamin A, 10000.00 IU; vitamin D3, 3000.00 IU; vitamin E, 30.00 IU; vitamin K, 2.20 mg; thiamin, 2.20 mg; riboflavin, 3.00 mg; niacin, 30.00 mg; vitamin B_{12}, 0.022 mg; biotin, 0.23 mg; niacin, 40.00 mg; pantothenic acid, 40.00 mg; folic acid, 1.00 mg; Mn, 120.00 mg; Zn, 100.00 mg; Fe, 120.00 mg; Cu, 80.00 mg; I, 0.45 mg; Se, 0.30 mg.

Table S2. The relative abundance of *Firmicutes, Bacteroidetes, Proteobacterial,* and *Tenericutes* in broilers treated with chlortetracycline and *Bacillus amyloliquefaciens TL* and in a control group on days 7, 14, 21, and 35.

Taxonomy	*Firmicutes*	*Bacteroidetes*	*Proteobacteria*	*Tenericutes*
A7	0.858311	0.005257	0.038977	0.065403
P7	0.889899	0.005861	0.016449	0.042215
C7	0.886309	0.000459	0.016678	0.051695
A14	0.859936	0.00643	0.0276	0.038805
P14	0.852375	0.003953	0.033928	0.044701
C14	0.757095	0.040313	0.070332	0.041214
A21	0.768345	0.014136	0.021959	0.040757
P21	0.767178	0.029265	0.039586	0.041946
C21	0.556751	0.23953	0.067086	0.040143
A35	0.71719	0.100204	0.036267	0.06729
P35	0.728244	0.101312	0.034155	0.041119
C35	0.606999	0.236127	0.03757	0.036152

‘A’ = Chlortetracycline group, ‘P’ = *Bacillus amyloliquefaciens* TL group, ‘C’ = control group

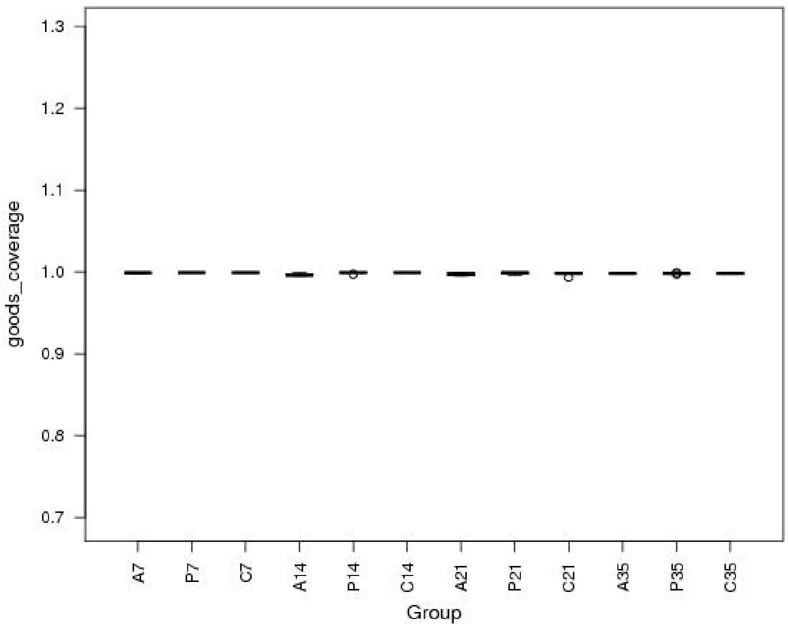

Figure S1. The goods_coverage index of the 80 broilers treated with Chlortetracycline and *Bacillus amyloliquefaciens TL* and in a control group on days 7, 14, 21, 35. 'A' = Chlortetracycline group, 'P' = *Bacillus amyloliquefaciens* TL group, 'C' = control group.

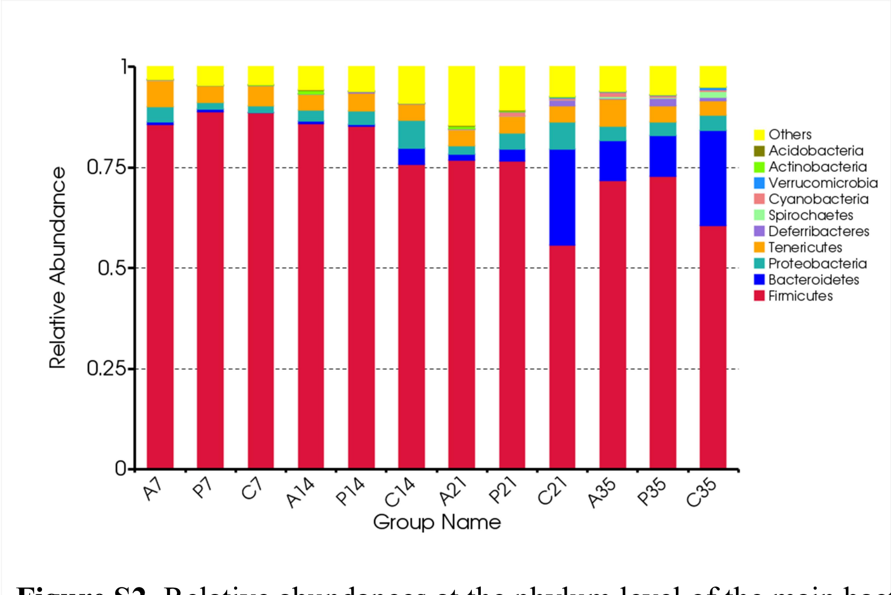

Figure S2. Relative abundances at the phylum level of the main bacteria found in the cecum of 80 broiler chicken treated with Chlortetracycline and *Bacillus amyloliquefaciens TL* and in a control group on days 7, 14, 21, 35. 'A' = Chlortetracycline group, 'P' = *Bacillus amyloliquefaciens* TL group, 'C' = control group.
